# Sustainable urban governance: how digital platforms shape whole-process youth participation intention and behavior

**DOI:** 10.3389/fpsyg.2026.1952419

**Published:** 2026-09-22

**Authors:** Yan Huang, Xinyue Ma, Da Gao, Kai Wu

**Affiliations:** 1 School of Management, Wuhan Institute of Technology, Wuhan, Hubei, China; 2 School of Marxism, Wuhan Institute of Technology, Wuhan, Hubei, China; 3 School of Law and Business, Wuhan Institute of Technology, Wuhan, Hubei, China

**Keywords:** cognition-affect-conation framework, digital platform empowerment, participation intention and behavior, sustainable urban governance, whole-process youth participation intention

## Abstract

**Introduction:**

As digital transformation empowers sustainable urban governance, digital whole-process democracy provides an institutional mechanism to mobilize youth participation and promote sustainable development at the grassroots level. However, existing research has not explained how digital platforms influence young people's participation intention and behavior. Drawing on the cognition-affect-conation (CAC) framework, technology acceptance model (TAM), and theory of planned behavior (TPB), this study constructs a parallel mediation model to explain how digital platforms shape whole-process youth participation intention and behavior.

**Method:**

This study employs a mixed-methods approach combining behavioral data, a questionnaire survey, and semi-structured interviews to examine whole-process youth participation on a digital platform. Behavioral data from the urban message board platform are collected first to provide empirical evidence of digital participation. In addition, a questionnaire survey is administered to test the impact of digital platforms' perceptions on youth participation intention. Semi-structured interviews are conducted to explore factors that facilitate the translation of participation intention into actual behavior.

**Results:**

The results show that (1) Digital platforms provide low-threshold participation channels that span the entire governance process. However, youth participation remains uneven. (2) Youth's perceived usefulness and ease of use positively influence whole-process youth participation intention throughout the entire sustainable urban governance. (3) Digital political trust and digital political efficacy play parallel mediating roles between digital platforms' perceptions and whole-process youth participation intention. (4) Digital literacy is a crucial factor in transforming whole-process youth participation intention into sustained and actual behavior. This study expands the theoretical boundaries of youth political participation and advances the sustainable modernization of urban governance.

**Discussion:**

These findings offer new insights into the discourse on the low-threshold effect in digital participation by clarifying the gap between digital channels and sustained participation. The study integrates the TAM and the TPB into the CAC framework, validating and extending the applicability of these theories to contexts of sustainable youth participation on digital platforms. Meanwhile, these results enrich the concept of perceived behavioral control within the TPB. This study provides a new research perspective on how government digital platforms, young people, and the social governance environment can work together to promote sustainable urban governance.

## 1 Introduction

Whole-process democracy is a political philosophy that emphasizes sustained, inclusive, and participatory decision-making, allowing stakeholders to actively engage in shaping policies and incentives that govern their interactions (Wang K. et al., 2025). Chinese youth, as the most energetic segment of society, are active participants throughout the democratic process (Vorderer et al., 2016). With rapid technological advances, digital platforms have dismantled traditional media gatekeeping and become essential channels for the continuous transmission of political information (Yang et al., 2025). Consequently, the arena for youth participation has expanded from physical to digital spaces (Lin et al., 2025). How youth utilize these platforms to engage in the whole-process of democratic governance is both a practical exploration of digital democracy and a critical theoretical issue for sustainable urban governance.

The theory of planned behavior (TPB) emphasizes the importance of volitional factors. It defines perceived behavioral control as an individual's evaluation of the ease or difficulty associated with performing a specific behavior (Ajzen, 1991). Building on this concept, perceived ease of use is defined as the degree to which a person believes that using a given technology requires no effort (Davis, 1989). The technology acceptance model (TAM) is a theoretical framework for predicting individuals' acceptance of new technologies and their subsequent usage behavior. According to this model, an individual's intention is the primary determinant of actual behavior (Davis, 1989; Yeh et al., 2024). The model further defines perceived usefulness as an individual's evaluation of the practical benefits that digital technology offers. This study develops a research model based on the cognition-affect-conation (CAC) framework, which posits that youth's perceived usefulness and ease of use would influence their affective states. These affective states then foster sustained political participation intention, which then translates into actual behavior.

Previous research has examined the role of digital technology from two complementary perspectives. On the government side, studies have explored how digital technology accelerates government transformation (Han and Zhang, 2024) and enhances governance capacity (Chen et al., 2023). On the citizen participation side, studies have focused on how digital technology facilitates political engagement by offering low-threshold opportunities (Hoffmann and Lutz, 2023; Boulianne, 2020) and connecting diverse stakeholders (Borrero and Borrero-Domínguez, 2024). Additionally, digitally enabled smart city governance promotes inclusiveness and sustainability by creating tangible public service participation experiences (Chang, 2026) and driving innovation in sustainable governance (Kang and Wang, 2020).

However, the literature on whole-process youth participation reveals several gaps. Most studies emphasize social media participation, which often lacks substantive policy impact (Waeterloos et al., 2023). In addition, young people are commonly viewed as ordinary citizens, while their unique agentic roles are overlooked. As a result, this limited perspective constrains strategies aimed at fostering youth civic participation (Tarman and Kilinc, 2023). Furthermore, existing research has yet to explore how digital technologies can support coherent youth participation throughout all stages of sustainable governance from a whole-process democracy perspective. Filling these gaps will highlight the proactive role of youth, refine the analytical framework for whole-process participation, and generate theoretical evidence that supports the interdisciplinary integration of whole-process democracy, youth participation, and sustainable urban governance.

Therefore, this study utilizes an integrative framework to analyze survey and behavioral data on youth participation. It aims to analyze how digital platforms influence whole-process participation intention and actual participation behaviors. Based on this, this study seeks to answer the following questions:

RQ1: Can digital technologies provide practical scenarios for sustained whole-process youth participation?
RQ2: How can we depict the landscape of whole-process youth participation on digital platforms?
RQ3: What factors influence whole-process youth participation intention and substantive behavior via the digital platform?

This study makes significant contributions to both theory and practice. First, it moves beyond a one-dimensional view of political participation. Drawing on the political philosophy of whole-process democracy, it develops a theoretical framework of youth digital participation that covers expression, interest negotiation, decision formation, decision implementation, and evaluation and oversight. This framework presents how digital technology supports youth participation in sustainable urban governance. Second, the study proposes an integrated framework combining the CAC framework, the TAM, and the TPB. This framework explains how young people's technological perceptions develop into actual political behavior. Focusing on official governance platforms, the study examines the sustainable political participation of Chinese youth and provides theoretical support for building youth-friendly cities in China. Finally, using a mixed-methods approach, the study shows how young people's perceptions of platform technology shape their political trust and efficiency, participation intentions, and actual behaviors. It therefore clarifies the psychological dynamics of youth participation throughout the whole-process and provides empirical evidence.

## 2 Literature review

### 2.1 Cognition-affect-conation framework

The CAC framework comprises cognition, affect, and conation (Rosenberg and Hovland, 1960). It is used to explain attitude formation, cognition refers to individuals' understanding and interpretation of information, affect denotes their feelings and evaluations, and conation reflects their intended actions and behavioral tendencies (Zhang et al., 2024; Xia et al., 2023; Tian et al., 2026). This framework is applied to examine how perceptions of new technologies influence attitudes or behaviors through affective pathways. For instance, Dai et al. (2020) found that perceived information overload on social media triggered emotional responses that shaped later behaviors. Huang et al. (2019) showed that middle-aged individuals' perceptions of health applications significantly affected their usage attitudes. Additionally, researchers have used this framework to study continued usage intentions toward digital media platforms (Zeng et al., 2023), short-video platforms (Mou et al., 2021), and online learning platforms (Khan et al., 2023).

### 2.2 Youth political participation in the digital era

Political participation encompasses the direct and indirect actions through which citizens seek to influence governmental decision-making. It includes institutional participation, non-institutional participation, and non-political activities (Theocharis et al., 2021; Bäck and Christensen, 2016). Government digital transformation has reshaped civic political participation by shifting traditional participation to sustained digital participation. Digital participation refers to the use of information technologies to enhance public engagement in decision-making and governance (Nurlinah et al., 2025). As young people assume a greater role in social governance, youth political participation has expanded. This concept is defined as the active participation of youth in democratic processes and the development of community responsibility (Borrero and Borrero-Domínguez, 2024). Such participation takes place through various channels, including social media use, online discourse, and community organizing. The growing adoption of digital platforms enables young people to articulate their demands and contribute to social and political change (Gaby, 2017; Loader et al., 2014).

Youth political participation faces three main challenges. First, the suspension of political participation institutions (Santi Amantini, 2025) and the lack of legal safeguards (Bani-Hani and Alhathloul, 2022) leave young people without public platforms for free expression, rational deliberation, and democratic dialogue, narrowing their channels for participation. Second, scarce social resources have pushed young people toward irrational involution, reflecting a growing trend of individualization (Constantin, 2024), which limits the formation of their public spirit (Liu and Zhang, 2025). Third, young people exhibit insufficient capacity for political participation, as evidenced by their inadequate cultural literacy regarding public issues, limited awareness of participation channels, weak critical thinking, and underdeveloped civic competencies (Myoung and Liou, 2022; Chia and Neoh, 2024; Munzir et al., 2025). Therefore, to promote sustainable urban governance, it is essential to clarify the role of digital platforms to investigate the mechanisms that influence whole-process youth participation.

### 2.3 Digital platform empowerment for sustainable urban governance

Sustainable urban governance refers to the institutional system and operational processes that reconcile current and future urban needs through inclusiveness and sustainability (Belizaire, 2026). In the digital era, achieving this goal depends on the coordinated development of governance systems and technological innovation. Digital platforms serve as infrastructures that integrate multi-source data, coordinate public services, and reshape public value. They are thus recognized as a key driver of modern urban governance transformation (Kozaman Aygün and Inal Çekiç, 2025). At the technological level, digital platforms integrate data-driven governance systems, thereby providing essential technical support for smart eco-city development (Bibri et al., 2024). At the institutional level, digital platforms reduce the costs of public participation, enabling citizens to engage in urban governance and expanding the democratic foundation (Doudoute and Garno, 2026).

Digital platforms have reduced participation barriers, enabling public involvement in governance processes (Belizaire, 2026). However, translating engagement into effective governance remains challenging. Falco and Kleinhans (2018) show that online participation is limited by digital literacy gaps and power structures, resulting in symbolic consultation. Therefore, advancing from formal to substantive participation and exploring long-term pathways for comprehensive participation are critical issues for sustainable urban governance.

## 3 Theoretical analysis and hypotheses

### 3.1 A theoretical framework of digital platform empowerment

Digital technologies are reshaping youth participation in sustainable urban governance. Digital platforms have responded to the core demands of political participation, making inclusive and routine participation feasible (Wang T. et al., 2025). They not only overcome the spatial-temporal barriers and knowledge gaps inherent in traditional participation but also transform youth engagement from scattered individual actions into a closed-loop process that spans all links of expression, interest negotiation, decision formation, decision implementation, and evaluation and oversight. Within long-term inclusive governance, digital platforms sustain youth integration into whole-process democratic practices in rural settings through information sharing and collaboration (Liu, 2025; Chen and Cheng, 2026). Digital technology supports the full procedural framework of youth participation while fostering their agency and public spirit. This provides a solid practical foundation for the resilient development of urban governance and the sustainable participation of citizens. Consequently, digital platforms serve as a sustainable intermediary that connects individual youth actions with broader governance structures, thus enabling a comprehensive and multidimensional approach to youth participation.

The expression of demands constitutes a critical entry point for whole-process democracy (Nie and Wang, 2022). Digital technologies have created online spaces for participation, enabling young people to express their demands. The consultation mechanisms built on digital platforms allow young people to exchange information and foster inclusive understanding in social governance processes, ensuring that decisions reflect public opinion. In democratic decision-making, platforms integrate diverse viewpoints, standardize procedures, and enhance responsiveness, thereby strengthening sustained youth participation (Chen and Cheng, 2026). Democratic management enables timely government feedback through platforms, deepening and broadening participation (Sanarico, 2026). Democratic oversight relies on platforms' data-collection functions and transparent interactive mechanisms to enhance the authenticity of supervision (Song and Xia, 2024; Shin et al., 2024).

Overall, digital platforms integrate participation processes into a coherent whole. They cultivate a long-term inclusive ecosystem for youth participation in both depth and breadth, advancing the vision of sustainable urban governance. Thus, this study develops a theoretical framework for digital platform empowerment to support whole-process youth participation, as shown in Figure 1.

**Figure 1:**
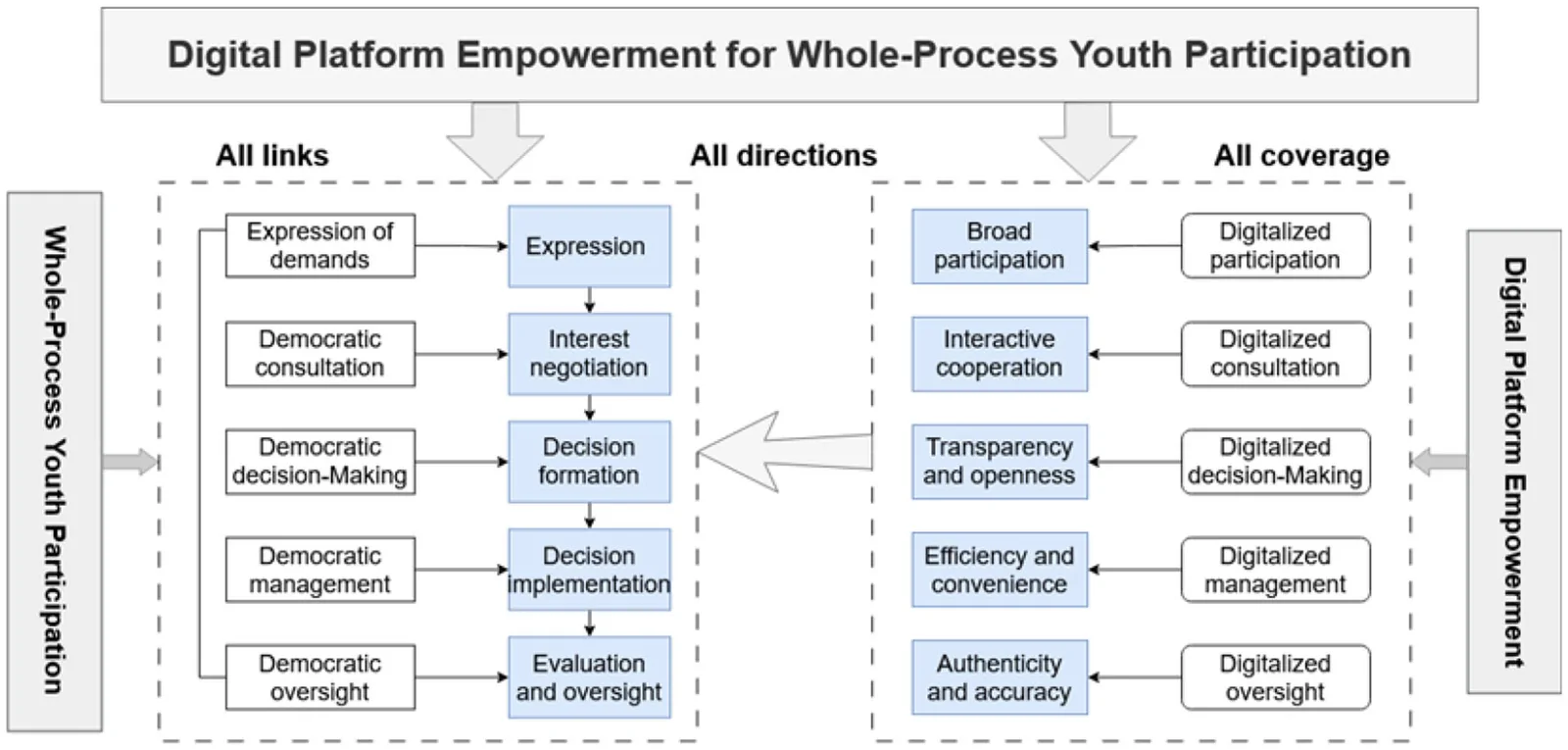
The theoretical framework of digital platform empowerment for whole-process youth participation.

### 3.2 The influence of digital platforms' perceptions on whole-process youth participation

By integrating the CAC, TPB, and TAM frameworks, this study suggests that whole-process youth participation intention is influenced by factors including digital platforms' perceptions, digital political trust, and digital political efficacy. Digital platforms' perceptions comprise perceived usefulness and perceived ease of use. This study conceptualizes these two dimensions as cognitive beliefs regarding digital platforms. Perceived usefulness refers to the benefits that young people expect to gain from using digital platforms, and perceived ease of use reflects the extent to which they find these platforms effortless and convenient (Davis, 1989). In the context of whole-process digital participation, young people's core concerns focus on whether digital participation realizes their rights and interests (Hatami et al., 2025). They also emphasize whether their individual demands are taken into account during decision-making (Rabe et al., 2026). In addition, they are concerned about whether such participation promotes social fairness, justice, and the oversight of public power (Wang et al., 2026). Positive outcomes achieved through digital platforms increase their participation intention. When a platform offers a user-friendly interface, comprehensive functionality, system stability, and other positive technical features, it can enhance users' participation intention (Yang and Lee, 2026). At the same time, perceived ease of use is closely associated with young people's usage behavior (German Ruiz-Herrera et al., 2023). The better young people understand the basic functions and operations of digital platforms, and the more convenient and easy they find them, the higher their acceptance of this mode of digital participation will be. As a result, their perceived usefulness of digital platforms will also increase. Therefore, this study posits that young people form participation intentions based on their perceived usefulness and perceived ease of use of digital platforms, and that perceived ease of use also enhances their perceived usefulness. Accordingly, the following research hypotheses are proposed:

*H1a:* Perceived usefulness positively affects whole-process youth participation intention.
*H1b:* Perceived ease of use positively affects whole-process youth participation intention.
*H1c:* Perceived ease of use positively affects perceived usefulness.

### 3.3 The mediating effects of digital political trust and digital political efficacy

Based on the CAC framework's logical chain, cognition influences behavioral intention through affective responses. Prior studies have confirmed that perceived usefulness and ease of use of digital technologies can enhance public officials' confidence and political efficacy (Jeilani et al., 2025; Karnsomdee, 2026). Both political trust and efficacy have been found to promote political participation intention (Wen et al., 2025; Munzir et al., 2025). Furthermore, from the citizen perspective, perceived usefulness of government websites positively affects participation, with political trust and efficacy serving as mediators (Lai and Beh, 2025; Hutahaean et al., 2023). In the context of this study, digital political trust and digital political efficacy are considered emotional factors. Digital political trust denotes their confidence in the digital government's capacity to perform effectively and deliver expected outcomes. Therefore, this study suggests that digital political trust acts as a key mediator, leading to the following hypotheses:

*H2a:* Digital platforms' perceptions positively affect digital political trust.
*H2b:* Digital political trust positively affects whole-process youth participation intention.
*H2c:* Digital political trust mediates the relationship between digital platforms' perceptions and whole-process youth participation intention.

Self-efficacy refers to individuals' beliefs about their ability to perform specific tasks (Bandura, 1977; Huang et al., 2025). Applied to the political domain, this concept suggests that political self-efficacy is an important factor shaping political participation (Kushin et al., 2022). In this study, digital political efficacy is defined as young people's emotional belief that they can use digital platforms to participate in political affairs and exert political influence. In the process of digital participation, enhanced perceptions of digital platforms broaden young people's exposure to political information (Amin et al., 2025). This access fosters the development of political efficacy, which leads to positive expectations of government performance. Consequently, young people believe their participation can impact policy formulation or implementation. This belief motivates active participation driven by self-actualization, political expression, and interest articulation, thereby enhancing their participation intention. Accordingly, the following hypotheses are proposed:

*H3a:* Digital platforms' perceptions positively affect digital political efficacy.
*H3b:* Digital political efficacy positively affects whole-process youth participation intention.
*H3c:* Digital political efficacy mediates the relationship between digital platforms' perceptions and whole-process youth participation intention.

In summary, this study develops a model of how digital platforms' perceptions, digital political trust, and digital political efficacy influence whole-process youth participation intention based on the integrated CAC framework, as shown in Figure 2.

**Figure 2:**
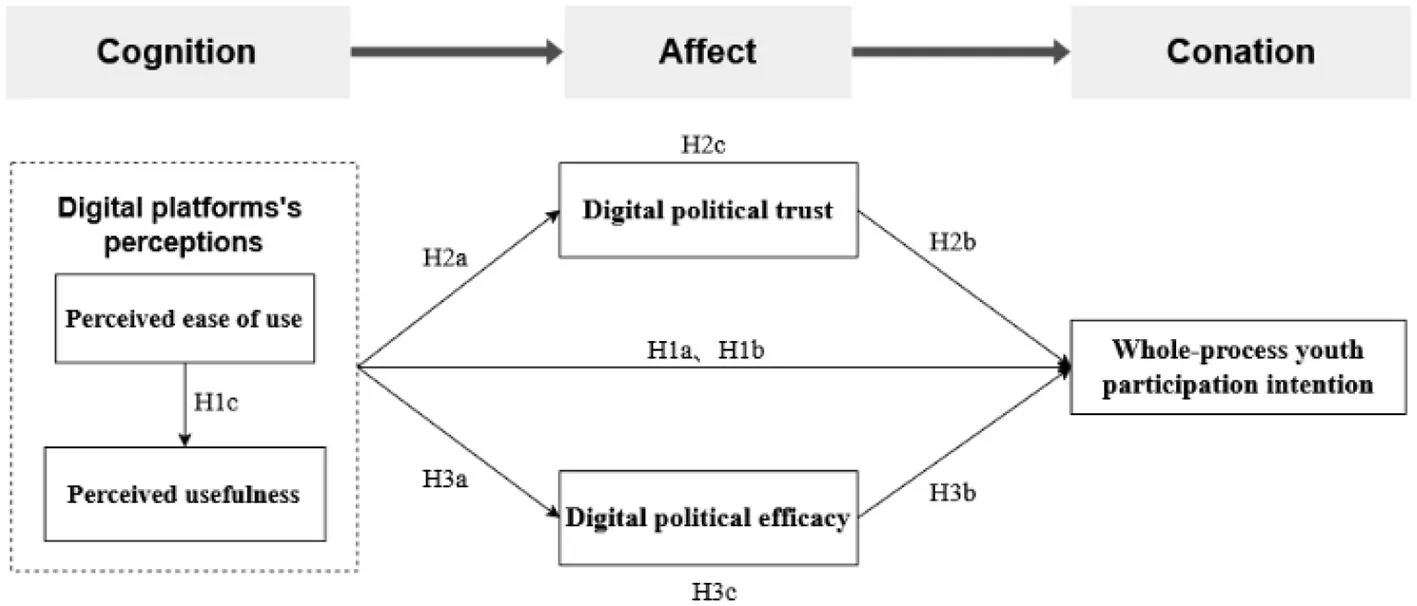
The model of the influence of digital platforms' perceptions on whole-process youth participation intention.

## 4 Research methodology

This study employs a mixed-methods approach combining behavioral data, a questionnaire survey, and semi-structured interviews to examine whole-process youth participation on a digital platform. Behavioral data from the urban message board platform are collected first to provide empirical evidence of digital participation. In addition, a questionnaire survey is administered to test the impact of digital platforms' perceptions on youth participation intention. Semi-structured interviews are conducted to explore factors that facilitate the translation of participation intention into actual behavior.

### 4.1 Behavioral data

#### 4.1.1 Case selection

This study focuses on the Wuhan urban message board platform, a key e-government platform consisting of five modules: “Leave a message,” “Hot topics,” “Deliberative decision-making,” “Inquiry on handling,” and “Satisfaction evaluation.” These modules correspond to the five stages of whole-process participation: expression, interest negotiation, decision formation, decision implementation, and evaluation and oversight. Unlike top-down government portals, this platform allows youth to express opinions on policies, rights, and obligations. Compared to social media, discussions are more focused and rational. The platform offers convenient and intelligent channels with high monthly activity, message volume, processing rates, and responsiveness. Youth can participate anytime via web or WeChat programs. Figure 3 illustrates the whole-process modules.

**Figure 3:**
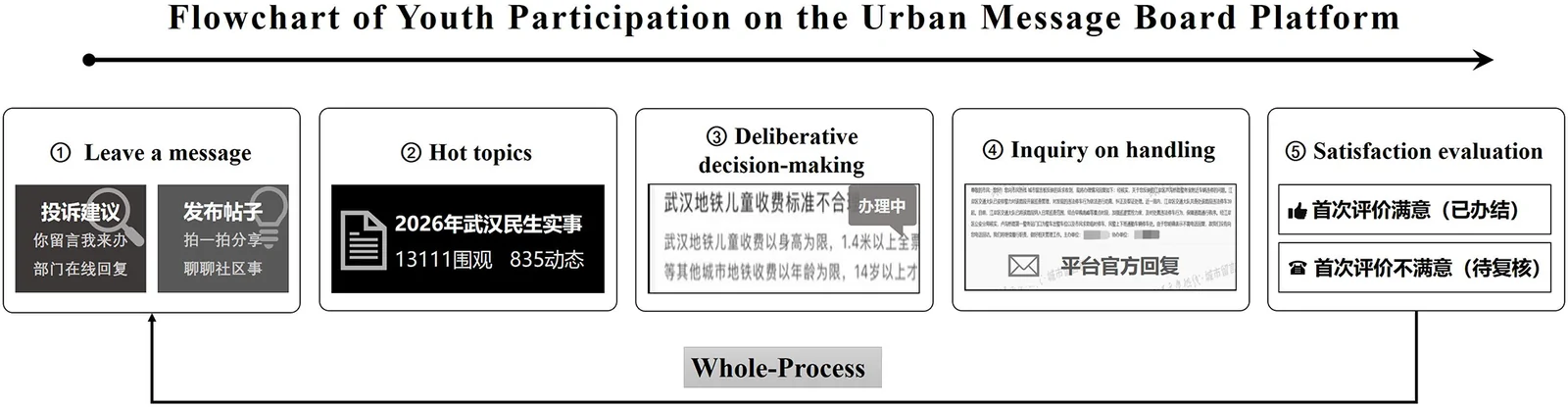
The whole-process module diagram of the urban message board platform. Reproduced with permission from the original poster. Licensed under liuyan.cjn.cn.

#### 4.1.2 Data extraction

Through institutional access granted by a collaboration with the Government Service Center, behavioral data are collected from users participating in the 15th Five-Year Plan suggestion-solicitation campaign between August 5 and September 30, 2025. Using a self-developed Python scraper, anonymized behavioral data are extracted, then manually coded and analyzed. After data cleaning, the dataset included 607,047 records across all five modules.

### 4.2 Questionnaire survey

#### 4.2.1 Sampling

From October 5 to November 26, 2025, a survey on whole-process youth digital participation was conducted using quota sampling based on administrative districts and youth population size in Wuhan (Neuman, 2011). The study distributed 6,445 questionnaires to young people via email, the Credamo platform, and paper surveys across all 13 county-level districts. After identifying and excluding invalid questionnaires characterized by rapid completion, non-standard answers, or logical inconsistencies, 5,310 valid questionnaires were retained. The questionnaire achieved an effective return rate of 82.4%. According to the 2025 Wuhan Statistical Yearbook, the 18–35 age group in the city comprises approximately 50.36% males and 49.64% females, with an average of 14.54 years of education. In our sample, males accounted for 50.09% and females for 49.91%, with an average education of 14.85 years, indicating strong representativeness.

#### 4.2.2 Instrument

A survey is conducted to test the proposed hypotheses. This study examines four variables: digital platforms' perceptions, digital political trust, digital political efficacy, and whole-process youth participation intention. All scales are adapted from validated instruments in authoritative journals and are revised to suit the study context. A five-point Likert scale (1 = strongly disagree, 5 = strongly agree) was used to specify the level of agreement with the statements.

#### 4.2.3 Variable measurement

The dependent variable is whole-process youth participation intention, referring to individuals' willingness to engage in expression, interest negotiation, decision formation, decision implementation, and evaluation and oversight. A screening question first identified respondents who were willing to use the Wuhan urban message board platform for urban governance participation. Subsequently, five items measured respondents' intention to participation at each stage. Respondents are asked: “If you are willing to use the digital platform to participate in community governance, to what extent would you participate in the following activities: (1) posting messages on the platform to express your personal demands; (2) initiating or joining discussions on issues of personal concern; (3) participating in discussions and voting on the resolution of issues; (4) following the government's responses to issue handling; and (5) promptly evaluating your satisfaction with the outcomes of issue handling.” Items for digital platforms' perceptions were adapted from ten-item scales from Davis (1989), including items such as “The digital platform makes it easier to access government information and communicate with others, enabling more participation” and “I find the digital platform easy to use.” Items for digital political trust were adapted from three-item scales from Sun et al. (2007), featuring statements such as “I believe digital platforms handle public affairs more quickly and effectively.” Items for digital political efficacy were adapted from three-item scales from Chen et al. (2019), including items such as “I think my participation via the digital platform can influence government decisions.” In addition, this study incorporated age, gender, income, education, and political affiliation as control variables.

### 4.3 Semi-structured interviews

To examine the translation of participation intention into actual behavior, this study matches survey responses with behavioral data from the urban message board platform. Respondents with high participation intention—defined as scores of 20–25 on the five-item scale—are first identified. Using the contact information provided in the survey, the research team contacts these users to confirm whether they had participated in the 15th Five-Year Plan suggestion-solicitation campaign via the platform. Those who had were invited to provide their user IDs. Ultimately, self-reported responses from 59 users were linked to their platform behavioral footprints. Tracking these users revealed a total of 3,122 valid behavioral records. Of these, 32 users participated in all five modules, while 16 agreed to participate in follow-up interviews. The interviewees included 12 males and four females, with an average age of 28.5 years. 93.75% held a bachelor's degree or higher. Four interviewees are members of the Communist Party of China, and their monthly income ranged from ¥2,200 to ¥20,000.

Semi-structured interviews are conducted with the 16 interviewees, focusing on three open-ended questions: (1) what factors facilitated your participation in the 15th Five-Year Plan suggestion-solicitation campaign via the urban message board platform? (2) Did you experience any technical difficulties or issues while using the platform? If so, what are they? (3) Do you intend to continue using the platform for urban governance participation in the future, and why? Each interview lasted 30–40 min, during which the research team employed probing questions to deepen the inquiry until thematic saturation was reached.

## 5 Results

### 5.1 Descriptive analysis of digital participation behavioral footprints

Using behavioral data from the digital platform, this study delineates the patterns of users' whole-process participation. Figure 4 shows user behavioral footprints over the 57-day observation period. The distribution of behavior throughout five modules is as follows: “Leave a message” accounted for 71.3%, “Hot topics” for 11.5%, “Deliberative decision-making” for 8.4%, “Inquiry on handling” for 5.9%, and “Satisfaction evaluation” for 2.9%. Participation in the “Leave a message” module peaked from August 25 to 27, with these 3 days comprising 33.3% of all records. This suggests that the platform's multi-channel publicity and extensive mobilization efforts following the campaign launch effectively promoted active public engagement. Peaks in the “Hot topics” and “Deliberative decision-making” modules occurred between September 5 and 8, accounting for 29.6 and 28.9% of their respective totals. In the “Inquiry on handling” and “Satisfaction evaluation” modules, activity notably increased on September 26 as the campaign approached its deadline, representing 27.9 and 26.6%.

**Figure 4:**
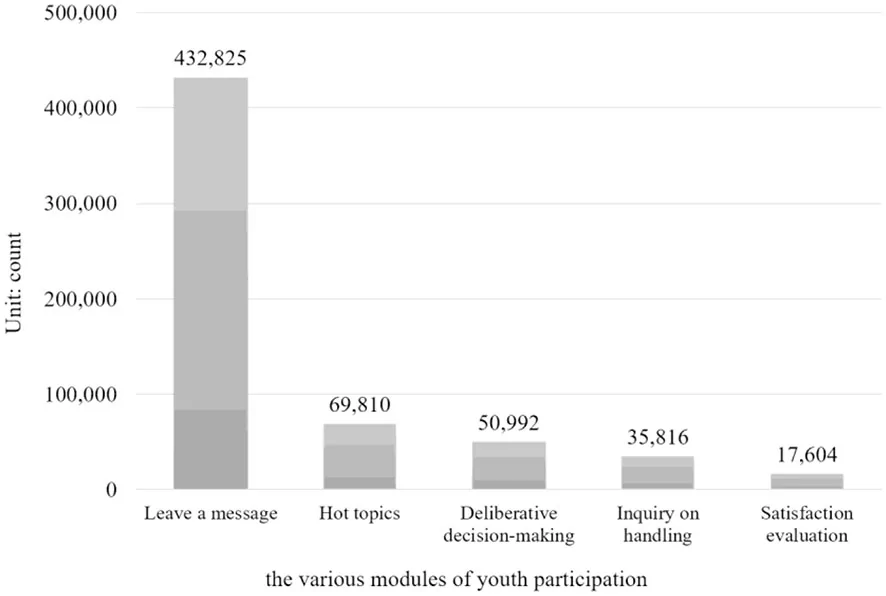
Behavioral data statistics.

Among the 432,825 behavioral records in the “Leave a message” module, 20.57% of users browsed the “Online services” window, and 2.08% viewed the “Message guidelines,” though neither group actively submitted messages. While these actions did not constitute a direct expression of demands, they reflect attentive participation. Additionally, 3.79% of users submitted messages that entered the review process, and 73.56% engaged by liking, commenting on, or reposting others' posts. The “Hot topics” module yielded 69,810 records, including 42.34% of users browsing hot topics, 20.81% posting comments, 1.88% initiating new topics, and 34.97% liking or replying to comments. These data indicate extensive interactive, participatory, and coconstructive deliberation. In the “Deliberative decision-making” module, 50,992 records showed that 44.73% of users viewed compiled posts under the “Netizens are asking” window, with others browsing government response information. Within the “Inquiry on handling” module's 35,816 records, 50.92% of users accessed the “Direct access to districts” window to monitor supervision progress and submitted relevant opinions. Finally, of the 17,604 records in the “Satisfaction evaluation” module, 68.56% of users rated their satisfaction with government responses and implementation outcomes, and 52.25% provided detailed reasons for their evaluations.

Statistical analysis of the behavioral data reveals an imbalance in digital participation, where attentive participation significantly surpasses substantive participation. Substantive participation occurs predominantly in the “Leave a message” and “Hot topics” modules. In contrast, participation in the “Deliberative decision-making,” “Inquiry on handling,” and “Satisfaction evaluation” modules remains limited. Despite this, the digital platform effectively supports users' real-time and targeted involvement throughout all governance stages. It establishes a closed-loop governance pathway for youth participation that links public opinion expression, government response, and the reexpression of public opinion.

### 5.2 Mechanisms of the influence of digital platforms' perceptions on whole-process youth participation intention

#### 5.2.1 Reliability, validity, and common method bias

This study used SPSS 27.0 and AMOS 28.0 (IBM Corporation, Armonk, New York, United States) to assess the reliability and validity of each scale, with results summarized in Table 1. Reliability analysis showed that Cronbach's α coefficients exceeded 0.8 for all variables, indicating strong internal consistency. Before confirmatory factor analysis (CFA), data suitability was confirmed with a Kaiser-Meyer-Olkin (KMO) value of 0.929 and a significant Bartlett's test of sphericity (*p* < 0.001). CFA results indicated good model fit with indices of χ^2^/df = 18.688, RMSEA = 0.058, CFI = 0.957, TLI = 0.950, and SRMR = 0.056. Convergent validity was supported by composite reliability (CR) values above 0.7 and average variance extracted (AVE) values exceeding 0.5 for all variables. Discriminant validity was confirmed as the square roots of AVE for each construct were greater than their inter-construct correlations. To address potential common method bias (CMB), Harman's single-factor test was conducted. The first principal factor explained 36.85% of the total variance, which is below the 40% threshold, indicating that CMB is unlikely to be a concern in this study.

**Table 1:** Validity and reliability assessment of the constructs.

Variables	Cronbach's α	CR	AVE	Discriminant validity				
				1	2	3	4	5
1. Perceived usefulness	0.910	0.911	0.674	**0.821**				
2. Perceived ease of use	0.908	0.908	0.664	0.553	**0.815**			
3. Digital political trust	0.839	0.839	0.635	0.425	0.513	**0.797**		
4. Digital political efficacy	0.807	0.808	0.583	0.548	0.461	0.268	**0.764**	
5. Whole-process youth participation intention	0.928	0.929	0.722	0.310	0.424	0.500	0.402	**0.850**

The bold values on the diagonal represent the square roots of the AVE.

#### 5.2.2 Descriptive statistics and correlations

Table 2 presents the means, standard deviations, and correlation coefficients for all variables. Perceived usefulness (*r* = 0.299, *p* < 0.01) and perceived ease of use (*r* = 0.388, *p* < 0.01) were positively correlated with the whole-process youth participation intention. These two perceptions were also positively correlated with each other (*r* = 0.520, *p* < 0.01). Perceived usefulness showed positive correlations with digital political trust (*r* = 0.388, *p* < 0.01) and digital political efficacy (*r* = 0.475, *p* < 0.01), while perceived ease of use correlated positively with digital political trust (*r* = 0.448, *p* < 0.01) and digital political efficacy (*r* = 0.405, *p* < 0.01). Both digital political trust (*r* = 0.441, *p* < 0.01) and digital political efficacy (*r* = 0.364, *p* < 0.01) were associated with participation intention. These results provide preliminary support for the proposed hypotheses. Multicollinearity was assessed through regression analysis and variance inflation factors (VIF), which ranged from 1.305 to 1.604 for all key variables, well below the threshold of 2, indicating no significant multicollinearity concerns.

**Table 2:** Descriptive statistics and correlation analysis.

Variables	1	2	3	4	5	6	7	8	9
1. Gender	—								
2. Political affiliation	0.076^*^	—							
3. Educational	0.026	0.035	—						
4. Income	0.028	0.029	0.176^**^	—					
5. Perceived usefulness	0.027	0.103^**^	0.145^**^	0.002	—				
6. Perceived ease of use	−0.005	0.062^*^	0.191^**^	0.067^*^	0.520^**^	—			
7. Digital political trust	0.006	0.088^**^	0.093^**^	0.043	0.388^**^	0.448^**^	—		
8. Digital political efficacy	−0.045	−0.032	0.075^*^	0.012	0.473^**^	0.405^**^	0.236^**^	—	
9. Whole-process youth participation intention	0.005	0.017	0.134^**^	−0.014	0.299^**^	0.388^**^	0.441^**^	0.364^**^	—
*M*	0.500	2.218	2.666	2.155	3.253	3.551	2.864	2.691	3.321
S.D	0.500	1.096	0.970	1.025	0.720	0.645	0.654	0.607	0.754

^*^p < 0.05.

^**^p < 0.01.

#### 5.2.3 Main effects and mediation effects testing

Multiple regression analysis was conducted to test the proposed hypotheses, with results detailed in Tables 3 and 4. In Table 3, Model 4 shows that perceived usefulness positively and significantly predicted the whole-process youth participation intention (β = 0.287, *p* < 0.001), supporting H1a. Model 3 indicates that perceived ease of use also had a significant positive effect on participation intention (β = 0.377, *p* < 0.001), supporting H1b. Additionally, Model 2 reveals that perceived ease of use significantly influenced perceived usefulness (β = 0.508, *p* < 0.001), confirming H1c.

**Table 3:** Results of main effects analysis.

Variables	Perceived usefulness of digital platforms		Whole-process youth participation intention	
	M1	M2	M3	M4
Gender	0.017	0.024	0.002	−0.009
Political affiliation	0.097	0.069	−0.008	−0.014
Educational	0.145	0.051	0.067	0.096
Income	−0.024	−0.035	−0.031	−0.016
Perceived usefulness		0.508^***^	0.377^***^	
Perceived ease of use				0.287^***^
*R* ^2^	0.031	0.279	0.155	0.098
Adjusted *R*^2^	0.028	0.276	0.151	0.094
*F*	8.568	81.774	38.798	23.066

^***^p < 0.001.

**Table 4:** Results of direct effects analysis.

Variables	Digital political trust		Digital political efficacy		Whole-process youth participation intention				
	M5	M6	M7	M8	M9	M10	M11	M12	M13
Gender	−0.003	−0.004	−0.044	−0.046	−0.005	−0.003	0.012	−0.003	0.005
Political affiliation	0.084	0.042	−0.031	−0.069	−0.020	−0.023	0.025	−0.034	−0.003
Educational	0.083	−0.006	0.077	−0.011	0.066	0.101^***^	0.110^***^	0.068	0.070
Income	0.044	0.045	−0.003	−0.011	−0.021	−0.042	−0.021	−0.036	−0.021
Digital platforms' perceptions		0.473^***^		0.501^***^	0.381^***^			0.224^***^	0.265^***^
Digital political trust						0.436^***^		0.333^***^	
Digital political efficacy							0.358^***^		0.226^***^
*R* ^2^	0.018	0.231	0.009	0.264	0.158	0.205	0.145	0.243	0.195
Adjusted *R*^2^	0.014	0.228	0.005	0.261	0.154	0.201	0.141	0.238	0.190
*F*	4.765	63.611	2.321	75.846	39.507	54.495	35.926	56.333	42.612

^***^p < 0.001.

To investigate whether the impact of digital platforms' perceptions on participation intention varied throughout the five stages, linear mixed model analyses were conducted. The results show that both the interaction between perceived usefulness and participation stage (*F*_(4, 6477)_ = 9.4, *p* < 0.001) and the interaction between perceived ease of use and participation stage (*F*_(4, 6477)_ = 15.15, *p* < 0.001) were significant. This indicates that the effects of perceived usefulness and perceived ease of use on participation intention differ depending on the stage of participation. As shown in Figure 5, the effect of perceived usefulness was significantly stronger in the early stages, such as the interest negotiation stage (β = 0.175), compared to the decision implementation stage (β = 0.093). Meanwhile, the effect of perceived ease of use was strongest during the decision formation stage (β = 0.445) and was significantly higher than in the evaluation and oversight stage (β = 0.352).

**Figure 5:**
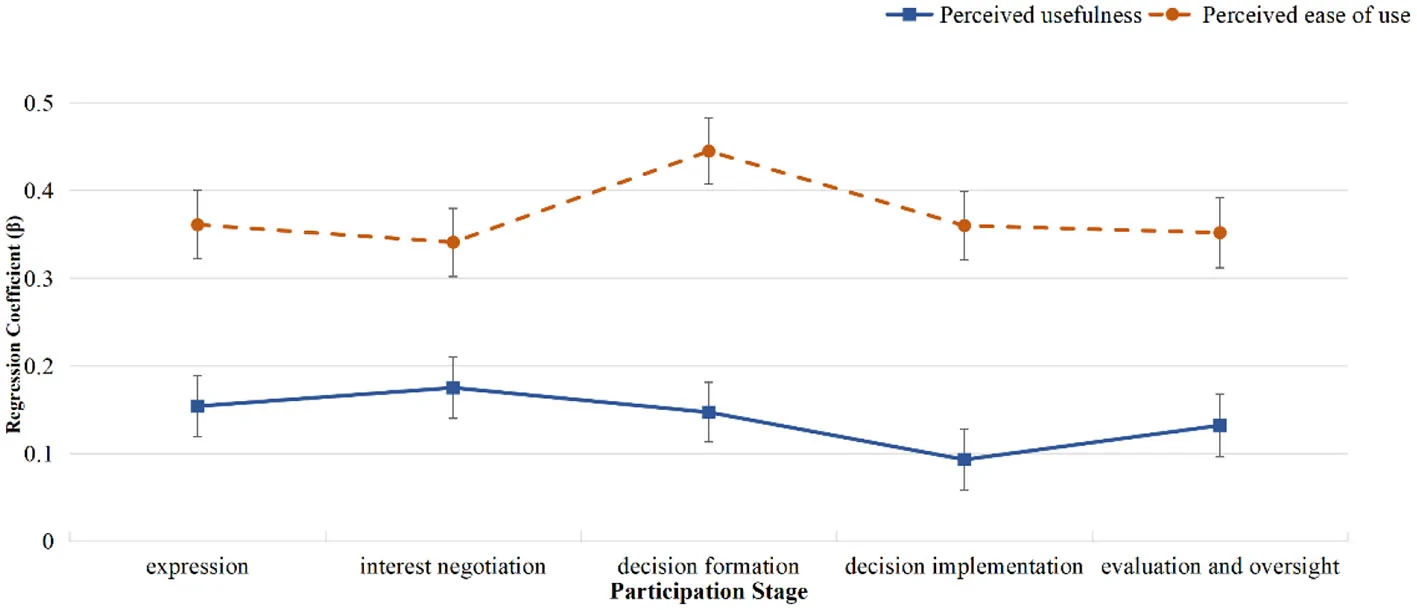
The relationship between perceived usefulness, perceived ease of use, and participation intention throughout participation stages.

Table 4 shows that digital platforms' perceptions positively affected digital political trust (β = 0.473, *p* < 0.001) and digital political efficacy (β = 0.501, *p* < 0.001) in Models 6 and 8, supporting H2a and H3a. Models 10 and 11 indicate that digital political trust (β = 0.436, *p* < 0.001) and digital political efficacy (β = 0.358, *p* < 0.001) both significantly predicted the whole-process youth participation intention, supporting H2b and H3b. In Models 12 and 13, when digital platforms' perceptions and each mediator were entered simultaneously, digital platforms' perceptions (β = 0.224, *p* < 0.001) and digital political trust (β = 0.333, *p* < 0.001), as well as digital platforms' perceptions (β = 0.265, *p* < 0.001) and digital political efficacy (β = 0.226, *p* < 0.001), all had significant positive effects on the whole-process youth participation intention. Compared to Model 9, after the inclusion of the two mediators separately, the coefficients of digital platforms' perceptions decreased but remained significant after including the mediators, indicating that digital political trust and digital political efficacy partially mediate the relationship between digital platforms' perceptions and the whole-process youth participation intention. Thus, H2c and H3c received preliminary support.

This study further tested the parallel mediation effects using the bootstrap method in the PROCESS macro with 5,000 resamples, as shown in Table 5. The results from the parallel mediation analysis show that the indirect effect of digital political trust on the relationship between digital platforms' perceptions and the whole-process youth participation intention was 0.1998, accounting for 40.3% of the total effect (*p* = 0.000). The indirect effect of digital political efficacy was 0.1448, representing 29.2% of the total effect (*p* = 0.000). The 95% bootstrap confidence intervals for these mediations were [0.1824, 0.2184] for digital political trust and [0.1276, 0.1613] for digital political efficacy. Neither interval included zero, indicating that both mediating effects were significant and providing further support for H2c and H3c. Meanwhile, a supplementary analysis tested the sequential mediation pathway from perception to digital political trust, then to digital political efficacy, and finally to participation intention. The results showed this sequential mediation was not statistically significant, indicating that digital political efficacy does not primarily develop through digital political trust. These findings thus reinforce the view that digital political trust and digital political efficacy function as related but distinct parallel mediators.

**Table 5:** Results of mediation effects analysis.

Items	Effect size	SE	95% Confidence interval (CI)		Proportion of indirect effect (%)
			LLCI	ULCI	
Total effect	0.4957	0.0160	0.4643	0.5270	
Direct effect	0.1511	0.0190	0.1139	0.1883	30.5
Path 1: digital platforms' perceptions → digital political trust → whole-process youth participation intention	0.1998	0.0090	0.1824	0.2184	40.3
Path 2: digital platforms' perceptions → digital political efficacy → whole-process youth participation intention	0.1448	0.0084	0.1276	0.1613	29.2
Contrasting indirect effects (1–2)	0.0550	0.0118	0.0317	0.0784	

### 5.3 Key transformation factors of substantive participation behavior

The interview findings revealed the underlying mechanisms driving the transformation of youth digital participation intention into sustained substantive behavior. Respondents' accounts indicated a clear cognition-attitude-behavior sequence. At the cognitive level, youth exhibited strong digital operational competence. One respondent noted ease in navigating the government platform, emphasizing digital literacy as essential for participation: “*I often use my mobile phone to participate in online activities, so I am familiar with this type of government platform. I know how to find the entry point, register and log in, edit text in the message box, upload pictures—none of these pose any difficulty for me.”* At the attitudinal level, participants demonstrated both trust in and awareness of risks associated with digital government platforms. One respondent stated, “*When I see that the activity is promoted by government websites and mainstream media, I feel reassured to participate. But sometimes I also worry about personal information leakage during participation, or that my intended meaning might be misunderstood by others, or that there may be other risks.”* At the conative level, youth demonstrated strong motivation for sustained engagement, believing that effective use of digital channels could address community issues. This reflects the role of digital cognitive ability and political efficacy in fostering participatory habits. One respondent stated, “*I hope my community can develop better and better. I also believe that the Internet has provided us with such a good channel today. As long as we make good use of it, the government will surely help us solve our problems. That is why I will continue using this platform in the future.”* Through systematic categorization and content analysis of the interview data, this study found that participation intention alone does not guarantee sustained substantive participation. Instead, the successful translation of intention into behavior depends on the combined influence of three dimensions: digital cognitive ability, digital application literacy, and digital risk awareness. These findings indicate that digital literacy serves as the key driver that enables youth participation intention to translate into ongoing substantive participation.

## 6 Discussion and conclusion

### 6.1 Discussions

Drawing upon the CAC framework, this study integrates the TPB and the TAM within a mixed-methods approach that combines behavioral data, a questionnaire survey, and semi-structured interviews. It examines the mechanisms influencing youth participation through the digital platform in governance and the factors facilitating the conversion of participation intention into substantive behavior. The main findings are: (1) digital platforms provide accessible participation channels throughout governance, but youth participation is uneven, with the highest engagement in expression and limited involvement in decision-making and oversight; (2) perceived usefulness and ease of use of digital platforms positively influence youth participation intention for sustainable urban governance; (3) digital political trust and efficacy mediate the relationship between digital platforms' perceptions and participation intention, forming a complete cognition–affect–conation pathway; (4) digital literacy is the key driver enabling youth participation intention to translate into sustained substantive participation.

#### 6.1.1 Digital platforms as the practical arena for whole-process youth participation

The behavioral footprint data show that digital platforms cover the five modules of expression, interest negotiation, decision formation, decision implementation, and evaluation and oversight, enabling whole-process youth participation. This finding supports Tai et al.'s (2024) view that digital technologies reduce traditional participation barriers and confirms Stier et al.'s (2022) conclusion regarding the mediating role of digital platforms in political information transmission. However, youth participation remains imbalanced, with attentive behaviors outweighing substantive engagement. Despite the technical provision of full participation channels, youth tend to engage more in expressive activities than in sustained supervisory roles. This pattern may stem from limited youth awareness of the later stages, suboptimal platform design, or underdeveloped feedback mechanisms that fail to motivate sustained engagement. These findings extend Ekström and Shehata's (2018) research on the low-threshold effect by highlighting the gap between accessible channels and substantive participation.

#### 6.1.2 Digital platforms' perceptions and whole-process youth participation intention

Survey results show that both perceived usefulness and perceived ease of use positively influence youth's whole-process participation intention, with ease of use having a stronger effect. This finding partly confirms the TAM (Davis, 1989) in political participation and indicates that in whole-process democracy, the convenience of digital technology more effectively stimulates initial participation intention than perceived utility. Further analysis reveals that perceived ease of use significantly enhances perceived usefulness, suggesting that when youth find platforms user-friendly, they recognize their practical value. This chain aligns with the TPB's view that perceived behavioral control affects intention (Ajzen, 1991) and supports Park et al.'s (2025) finding that ease of use improves user utility.

#### 6.1.3 The affective mediation of digital political trust and digital political efficacy

Based on the CAC framework, this study confirms that digital political trust and efficacy mediate the relationship between digital platforms' perceptions and whole-process youth participation intention. Perceived usefulness and ease of use first foster digital political trust and efficacy, which then lead to participation intention, supporting the cognition-affect-conation chain. Unlike previous studies, this research integrates the CAC framework with political trust and efficacy to build a comprehensive model for whole-process democracy. It also differentiates the two affective pathways, finding that political trust has a stronger mediating effect than political efficacy. This may reflect the importance of political trust in the Chinese governance context, consistent with Li and Yu (2023). Qualitative interview data, including statements like “the government will surely help us solve our problems,” further support the trust pathway.

#### 6.1.4 Digital literacy and youth substantive participation

Analysis of behavioral footprints on the digital platform indicates that a significant proportion of users limit their engagement to browsing or liking activities, without progressing to meaningful participation. Findings from a subsequent interview study highlight digital literacy as a crucial factor in transforming youths' intentions for comprehensive participation into actual substantive behaviors. This digital literacy encompasses three key dimensions: digital cognitive ability, digital application literacy, and digital risk awareness. These results suggest that within the context of whole-process digital participation, perceived behavioral control extends beyond individuals' subjective assessment of digital technology usability. It also includes their objectively demonstrated operational skills and their capacity to manage digital risks. This insight thus broadens the conceptual scope of the behavioral control construct within the TPB.

### 6.2 Theoretical contributions

The theoretical contributions of this paper are as follows.

First, this study advances the understanding of the low-threshold effect in digital political participation. Although existing literature generally posits that low-threshold digital participation channels promote broad engagement, the findings presented here indicate that young people's participation predominantly remains limited to low-cost activities such as expressive acts. In contrast, there is relatively minimal substantive participation in critical phases such as decision-making, management, and oversight. These findings offer new insights into the ongoing discourse on the low-threshold effect in digital participation by clarifying the gap between accessible digital channels and sustained engagement.

Second, this study integrates the TAM and the TPB into the CAC framework, thereby validating and extending the applicability of these theories to contexts of sustainable youth participation on digital platforms. The findings clarify the predictive effects of perceived ease of use and perceived usefulness on young people's intentions to participate throughout the entire governance process. They also demonstrate the positive relationship between perceived ease of use and perceived usefulness. Furthermore, by incorporating politically relevant affective variables, including digital political trust and digital political efficacy, into the CAC pathway, this study develops an integrated model for explaining young people's whole-process participation on digital platforms. This model helps address the theoretical fragmentation between technology acceptance research and political participation research. It also demonstrates the explanatory value of TAM and TPB in the context of public governance and civic participation.

Third, this study enriches the concept of perceived behavioral control within the TPB. Previous research generally defines perceived behavioral control as an individual's subjective assessment of the ease or difficulty of performing a given behavior. However, this study identifies digital literacy as a key condition through which young people's participation intentions are translated into substantive behaviors. Accordingly, it extends perceived behavioral control beyond subjective judgments about the difficulty of participation to encompass both subjective perceptions and objective digital literacy. This expanded conceptualization offers a more comprehensive explanation for the gap between young people's intentions to participate and their actual participation behavior in digital governance.

### 6.3 Practical implications

At the governmental level, authorities should continue strengthening digital platform development to build a robust technological foundation for sustainable urban governance. Specifically, governments need to enhance digital infrastructure and promote deep integration of digital platforms with social governance processes. This integration can facilitate the transformation of governance models toward more decentralized structures, thereby improving the efficiency and accessibility of whole-process participation. Additionally, governments should optimize the user experience of digital platforms to consistently reinforce youths' digital political trust and efficacy. Furthermore, by using digital platforms, governments ought to institutionalize participation channels through the systematic incorporation of youth suggestions and the provision of substantive feedback. Thus, this approach can expand institutional capacity for youth participation, enabling it to evolve from sporadic demands into routine practice and thereby generating endogenous drivers for sustainable governance.

At the youth level, enhancing digital literacy should be a key strategy for advancing governance modernization and building a sustainable social governance system. Given the ongoing digital governance transformation, youth are encouraged to strengthen their public responsibility and use digital platforms to engage in grassroots public affairs, including issue communication, deliberation, and decision-making, thereby shifting from peripheral observers to central actors. Also, as a generation raised in a highly digitalized environment, they should use their technological competencies to participate in online discussions and other governance activities in an orderly, effective, and comprehensive manner. Therefore, through sustained participation, they can accumulate experience and develop capabilities, fostering the continuous growth of citizen sustainability across generations.

At the societal level, collaborative efforts are needed to cultivate a supportive participation ecosystem and establish a long-term, inclusive environment for youth. Sustainable urban governance cannot rely solely on two-way interactions between government and youth. It also requires a social environment that features cultural identity and public spirit. Digital media should be used in ways that resonate with youth to create a public opinion climate that encourages active participation. Mass media must be utilized to build a multi-channel, multidimensional communication framework that objectively explains and widely disseminates the logic, boundaries, and risks of digital social governance. This will enhance youths' rational understanding, thus guiding them toward orderly and effective involvement. Lastly, social media platforms with large youth user bases should be harnessed to offer everyday digital spaces for youth participation. These platforms should not only facilitate institutionalized communication channels connecting youth and government, but also promote internal connectivity and mutual support among youth, embedding participation within their social networks and lifestyles. Ultimately, this will help form a sustainable, long-term, and inclusive participation ecosystem for urban governance with youth as principal actors.

### 6.4 Research limitations and future directions

This study has several limitations. First, the behavioral data were obtained from a single digital platform over a relatively short time period, and thus, the findings require validation through cross-regional and cross-platform comparative studies. Second, the questionnaire survey utilized a cross-sectional design; future research could be enhanced by incorporating longitudinal tracking or experimental approaches. Third, while this study explored the mediating effects of digital political trust and digital political efficacy, other potentially relevant variables remain unexamined. Subsequent research might consider including factors such as digital social norms and peer influence. Fourth, the small interview sample and the exclusion of individuals with high participation intentions but no corresponding behavior may have introduced survivorship bias. Future research should include such users to identify the barriers that hinder the translation of intentions into action. It should also examine the constraints faced by other groups to reveal multiple configurational pathways.

## Data Availability

The original contributions presented in the study are included in the article/supplementary material, further inquiries can be directed to the corresponding author/s.
